# Neuroligin 4 regulates synaptic growth via the bone morphogenetic protein (BMP) signaling pathway at the *Drosophila* neuromuscular junction

**DOI:** 10.1074/jbc.M117.810242

**Published:** 2017-09-14

**Authors:** Xinwang Zhang, Menglong Rui, Guangmin Gan, Cong Huang, Jukang Yi, Huihui Lv, Wei Xie

**Affiliations:** From the ‡ Institute of Life Sciences, the Collaborative Innovation Center for Brain Science, Southeast University, Nanjing, Jiangsu 210096, China,; ¶ Key Laboratory of Developmental Genes and Human Disease, Jiangsu Co-innovation Center of Neuroregeneration, Southeast University, Nanjing, Jiangsu 210096, China, and; the § Department of Biology, Basic Medical School of Shanxi Medical University, Taiyuan, Shanxi 030001, China

**Keywords:** bone morphogenetic protein (BMP), development, Drosophila, neurodevelopment, synaptic plasticity, NMJ, Neuroligin 4

## Abstract

The neuroligin (Nlg) family of neural cell adhesion molecules is thought to be required for synapse formation and development and has been linked to the development of autism spectrum disorders in humans. In *Drosophila melanogaster*, mutations in the *neuroligin 1–3* genes have been reported to induce synapse developmental defects at neuromuscular junctions (NMJs), but the role of *neuroligin 4* (*dnlg4*) in synapse development has not been determined. Here, we report that the *Drosophila* neuroligin 4 (DNlg4) is different from DNlg1–3 in that it presynaptically regulates NMJ synapse development. Loss of *dnlg4* results in reduced growth of NMJs with fewer synaptic boutons. The morphological defects caused by *dnlg4* mutant are associated with a corresponding decrease in synaptic transmission efficacy. All of these defects could only be rescued when DNlg4 was expressed in the presynapse of NMJs. To understand the basis of DNlg4 function, we looked for genetic interactions and found connections with the components of the bone morphogenetic protein (BMP) signaling pathway. Immunostaining and Western blot analyses demonstrated that the regulation of NMJ growth by DNlg4 was due to the positive modulation of BMP signaling by DNlg4. Specifically, BMP type I receptor thickvein (Tkv) abundance was reduced in *dnlg4* mutants, and immunoprecipitation assays showed that DNlg4 and Tkv physically interacted *in vivo*. Our study demonstrates that DNlg4 presynaptically regulates neuromuscular synaptic growth via the BMP signaling pathway by modulating Tkv.

## Introduction

The formation, development, and plasticity of synapses are critical for the construction of neural circuits, and the *Drosophila* larval neuromuscular junction (NMJ)^2^ is an ideal model system to dissect these processes (1). In the past few decades, several subcellular events and signaling pathways have been reported to be involved in regulating synaptic growth at *Drosophila* NMJs, such as local actin assembly, endocytosis, ubiquitin-mediated protein degradation, the wingless pathway, and the bone morphogenetic protein (BMP) pathway (1–6). Among these, BMP signaling is thought to be a major retrograde pathway that promotes the synaptic growth of NMJs (1, 2, 7).

At the *Drosophila* NMJ, the BMP homolog glass bottom boat (Gbb) is released by muscle cells and binds to the presynaptic type II BMP receptor wishful thinking (Wit). Wit is a constitutively active serine/threonine kinase and, upon binding to Gbb, forms a complex with the type I BMP receptor thickvein (Tkv) or saxophone (Sax), which results in their activation by phosphorylation. The activated type I receptor subsequently phosphorylates the downstream R-Smad protein mothers against decapentaplegic (Mad). Phosphorylated Mad (pMad) then binds to the co-Smad medea (Med). This complex translocates to the nucleus of motoneurons to activate or repress the transcription of target genes required for NMJ growth (1, 3, 4). Mutation of any component in the BMP signaling pathway results in a striking deficiency of NMJ growth (8–12). In addition, many molecules are reported to affect NMJ growth by negatively regulating BMP signaling at different points in the pathway (2, 12–20). Here, we report that *Drosophila* neuroligin 4 (DNlg4), a trans-synaptic adhesion protein, acts as a positive regulator of BMP signaling to regulate NMJ growth.

Neuroligins (Nlgs) were initially reported to be the postsynaptic ligands of the presynaptic adhesion proteins neurexins (Nrxs) (21–23), and loss of function of Nlgs in humans is thought to be associated with several mental disorders, including autism and schizophrenia (24–27). Nlgs are an evolutionarily conserved family of proteins encoded by four independent genes in rodents and five independent genes in humans (21, 28). Nlgs have been reported to induce synapse assembly by co-cultured neurons when expressed in nonneuronal cells, and overexpression of Nlgs in neurons increases synapse density (29–35). These *in vitro* cell culture studies suggest a role of Nlgs in inducing the formation of synaptic contacts. However, an *in vivo* study showed, despite severe defects in synaptic transmission, that there was no alteration of synapse number in neurons from *nlg1–3* triple knock-out mice (36). Similarly, loss of Nlg1 specifically in the hippocampus or amygdala did not alter the synapse number (37, 38), suggesting that the role of Nlgs is not to trigger the initial synapse formation. Rather, it is more likely that upon binding to Nrx, Nlg functions in maturation of nascent synapses, including differentiation and stabilization by recruiting scaffolding proteins, postsynaptic receptors, and signaling proteins (35, 36, 39–41).

In *Drosophila*, four Nlgs have been identified (42, 43). Mutations in *dnlg1–3* result in defective synapse differentiation that is primarily characterized by abnormal protein levels or the ectopic postsynaptic localization of glutamate receptors in larval NMJs. In addition, loss of DNlg1–3 separately leads to impairment in NMJ synapse development, as indicated by abnormal synaptic bouton number (43–46), but the precise underlying mechanism is poorly understood. A recent study showed that flies with a *dnlg4* mutation exhibit an autism-related phenotype of behavioral inflexibility, as indicated by impaired reversal learning (47). DNlg4 also regulates sleep by recruiting the GABA receptor to clock neurons and thus modulating GABA transmission (42), which suggests a role of DNlg4 in synapse differentiation. However, potential molecular mechanisms underlying the behavioral defects caused by *dnlg4* mutation and the role of DNlg4 in synapse development have not been reported.

Here, we reported the generation of an independent null allele of *dnlg4* and characterized the role of DNlg4 in neuromuscular synaptic growth. We showed that loss of DNlg4 led to impaired NMJ synapse growth, as indicated by decreased synaptic bouton numbers and increased bouton size. Presynaptic knockdown of DNlg4 mimicked these phenotypes. These morphological abnormalities in *dnlg4* mutants led to corresponding impairment in synaptic transmission efficacy. Unexpectedly, all of these defects were only rescued when DNlg4 was expressed in presynaptic, instead of postsynaptic, areas of NMJs. We next found that DNlg4 genetically interacted with components of the BMP pathway and that the presynaptic BMP signaling at NMJs was decreased in *dnlg4* mutants. We further observed a reduction of the BMP type I receptor Tkv in *dnlg4* mutants and demonstrated that DNlg4 physically interacted with Tkv *in vivo*. Altogether, our study revealed that DNlg4 regulated neuromuscular synaptic growth by positively modulating BMP signaling though Tkv.

## Results

### DNlg4 is predominantly expressed in central neurons and is concentrated at glutamatergic NMJs

The *dnlg4* (CG34139) gene is located on the right arm of the third chromosome at cytological position 92D5-8 and contains 16 exons and 15 introns. It encodes a protein of 1281 amino acids with a predicted molecular mass of 140 kDa. The DNlg4 protein is predicted to have three typical distinct regions similar to the typical structure of mammalian Nlgs: an extracellular region containing an N-terminal signal peptide and an inactive acetylcholinesterase-like domain, a single transmembrane domain, and a cytoplasmic region with a PDZ-binding motif.

**Figure 1.**
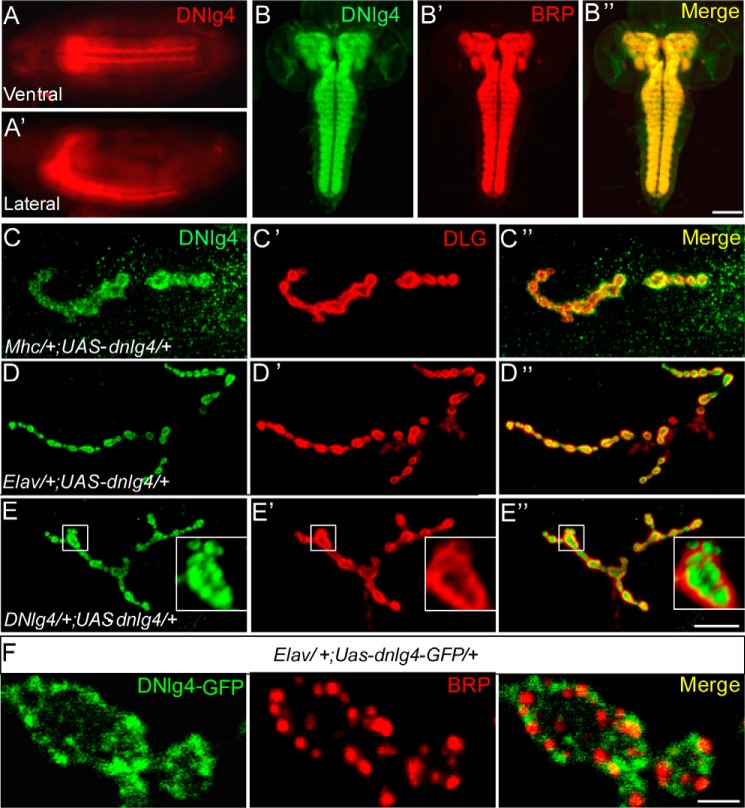
**DNlg4 is predominantly expressed in central neurons and concentrated at glutamatergic NMJs.** *A* and *A*′, lateral (*A*) and ventral (*A*′) views of stage 16 embryos from WT samples stained with an anti-DNlg4 antibody showing DNlg4 distributes in the CNS. *B–B*″, the third-instar larval brain stained for DNlg4 (*green*) and BRP (*red*), showing that DNlg4 distributes in the brain and ventral nerve cord (*VNC*) and is concentrated at the neuropil region. *C–D*″, the type Ib boutons of glutamatergic NMJ from flies overexpressing DNlg4 in muscle cells (*C–C*″) and neurons (*D–D*″) co-stained with anti-DNlg4 antibody (*green*) and anti-DLG antibody (*red*), showing that exogenous DNlg4 were concentrated in synaptic boutons of NMJs. *E–E*″, the type Ib boutons of NMJs from *dnlg4*-overexpressing flies driven by the DNlg4-Gal4 driver, co-stained with anti-DNlg4 and anti-DLG antibodies showing that DNlg4 was localized in the presynapse of NMJs. *F*, type Ib boutons of NMJs from flies overexpressing *UAS-dnlg4-gfp* in neurons stained with anti-BRP antibody (*red*); GFP is the spontaneous green fluorescence, showing that presynaptic DNlg4 is adjacent to active zones within synaptic boutons. *Scale bars*, 100 μm (*B–B*″), 20 μm (*C–E*″), and 1 μm (*F*).

To determine the expression pattern of endogenous DNlg4, we first performed embryonic immunostaining in WT flies with an antibody against the C terminus of DNlg4. DNlg4 was predominantly restricted to the brain and the ventral nerve cord (VNC) at embryonic stages 14–16 (Fig. 1, *A* and *A*′). In third-instar larvae, DNlg4 accumulated in the brain and VNC, as indicated by co-staining with antibodies against DNlg4 and Bruchpilot (BRP) (a presynaptic active zone marker molecule), and it was especially concentrated at the neuropil, where the synapses of neurons aggregate (Fig. 1, *B–B*″). We next sought to determine the distribution of DNlg4 at NMJs by immunostaining the larval body wall muscle with antibodies against DNlg4 and discs large (DLG) (a postsynaptic scaffolding protein and marker of type I boutons). However, we were unable to detect any immunoreactivity for endogenous DNlg4 at NMJs, perhaps because the protein level was too low to be detected or because the affinity of our antibody was not sensitive enough. We therefore overexpressed the full-length *dnlg4* cDNA in a WT background using the muscle-specific Mhc-Gal4 driver and the pan-neuronal Elav-Gal4 driver. In the lines in which DNlg4 was overexpressed in muscles, DNlg4 immunoreactivity accumulated at NMJs, but with a non-uniform distribution at distinct type I synaptic boutons (Fig. 1, *C–C*″). In contrast, the exogenous DNlg4 that was overexpressed in neurons exhibited a uniform distribution in type I boutons of NMJs, indicating that DNlg4 expressed in the neurons could be transferred to NMJs and could concentrate at synaptic boutons (Fig. 1, *D–D*″). This result suggested that DNlg4 was localized at the presynapses of NMJs. In most cases, presynaptic molecules in neurons tend to localize to presynaptic areas when they are overexpressed. We observed good presynaptic distribution of synaptotagmin (SYT; tagged by GFP), a well-known presynaptic molecule, at NMJs when it was overexpressed in motoneurons by a motoneuron-specific OK6-Gal4 driver. We also observed a presynaptic distribution of DNlg4-GFP at NMJs when we expressed a UAS-*dnlg4*-gfp cDNA using the Gal4 driver (supplemental Fig. S1, *A–B*′). However, when we overexpressed the postsynaptic molecule Dscam (48) (tagged by GFP) using this driver, only a very low Dscam signal could be detected at presynaptic boutons of NMJs, and most accumulated at the axons (supplemental Fig. S1, *C–C*″). These results suggested that the overexpressed DNlg4 at presynaptic boutons of NMJs was not an ectopic distribution caused by protein overexpression. More likely, DNlg4 was localized in the synaptic boutons of NMJs as a presynaptic molecule. Based on the above results, DNlg4 may exist in both the presynapse and postsynapse of NMJs. To further confirm the distribution of DNlg4 in synapses of NMJs, we generated a DNlg4-Gal4 line by inserting an ∼6-kb genomic sequence flanking the transcription start site of the *dnlg4* gene (which included the core promoter regions of the *dnlg4* gene) into the pPT-Gal4 vector and used it to drive UAS-*dnlg4* or UAS-*dnlg4*-gfp to mimic the expression of endogenous DNlg4. The DNlg4-GFP that was expressed by the DNlg4-Gal4 driver displayed an expression pattern similar to that of endogenous DNlg4 in larval brain, distributing in brain and VNC regions and concentrating in neuropils (supplemental Fig. S2*A*). In the peripheral nervous system, overexpressed DNlg4 distributed in synaptic boutons of NMJs. High-magnification confocal images showed that the DNlg4 and DLG were located at different regions of the bouton. Major DNlg4 signals were surrounded by DLG, and only a few DNlg4 signals overlapped with DLG in their interface (Fig. 1, *E–E*″). This result suggested that DNlg4 predominantly localized in presynaptic areas of boutons at NMJs. Overall, these results indicated that DNlg4 was predominantly expressed in the central neurons and was concentrated in presynaptic boutons of NMJs. However, postsynaptic localization of DNlg4 at NMJs could not be excluded.

To further define the precise subcellular localization of DNlg4 within synaptic boutons, we compared the position of DNlg4 with the presynaptic active zone (visualized with anti-BRP antibody) at NMJs by driving DNlg4-GFP with the Elav-Gal4 driver. DNlg4-GFP did not have a uniform distribution within a single bouton but instead displayed discontinuous patches (Fig. 1*F*). These DNlg4 patches did not overlap with BRP and instead localized adjacent to BRP, suggesting that the presynaptic DNlg4 was not localized in active zones but was adjacent to active zones in synaptic boutons of NMJs.

### The dnlg4 mutants display poor growth of NMJs and impaired synaptic ultrastructure at NMJs

**Figure 2.**
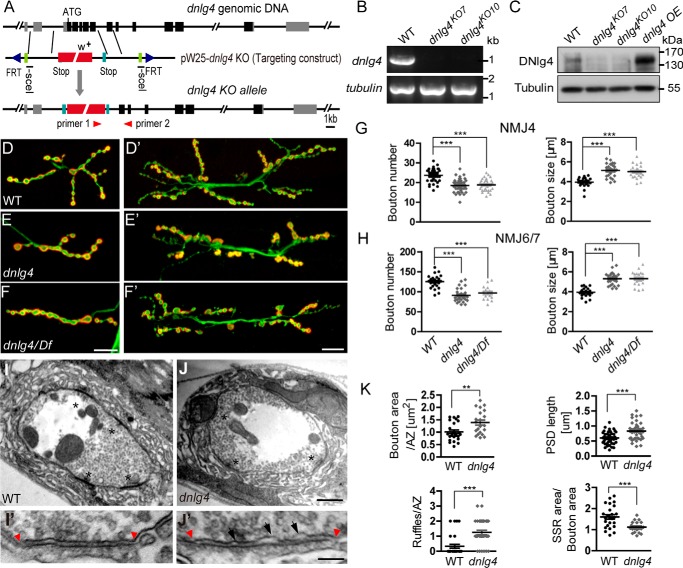
**The *dnlg4* mutants display poor growth of NMJs and impaired synaptic ultrastructure at NMJs.** *A*, the structure of *dnlg4* genomic DNA, the targeting construct, and the targeted locus. The positions of primers used for PCR screening of knock-out flies are shown at the *bottom. B*, RT-PCR analyses of total mRNA extracted from WT, *dnlg4^KO7^*, and *dnlg4^KO10^* flies with *dnlg4* primers, showing the presence of *dnlg4* mRNA in WT flies but not in either *dnlg4* knock-out mutant. *C*, Western blot analyses of larval brain extracts using anti-DNlg4 antibody showing the presence of DNlg4 protein (predicted to be ∼140 kDa) in WT and DNlg4-overexpressing flies driven by the Elav-Gal4 driver but absent in both mutant flies. *D–F*′, representative morphology of NMJ4 (*D–F*) and NMJ6/7 (*D*′–*F*′) double-stained for HRP and DLG in WT, *dnlg4*, and *dnlg4/Df. G* and *H*, quantification of bouton number and bouton size at NMJ4 (*G*) and NMJ6/7 (*H*) in the indicated genotypes. *I–J*′, representative transmission electron microscope micrographs of synaptic bouton from the WT (*I* and *I*′) and *dnlg4* mutant lines (*J* and *J*′), showing the synaptic boutons, the presynaptic AZs (*asterisks*), the PSD (between *two red arrowheads*), and the ruffles in presynapse (*black arrow*). *K*, quantification for bouton area per AZ, average single PSD length, the relative SSR area per bouton, and the ruffles per AZ in the WT and *dnlg4* mutant lines. For all comparisons, the Mann-Whitney test was used. ***, *p* < 0.001; **, *p* < 0.01. *Scale bar*, 20 μm (*D–F*″), 500 nm (*I* and *J*), and 100 nm (*I*′ and *J*′).

To address the *in vivo* functions of DNlg4, we generated a *dnlg4* mutant by gene targeting using the ends-out method (49). In brief, homologous recombination between the endogenous *dnlg4* locus and a donor DNA consisting of a mini-white marker gene flanked by 3.0- and 3.3-kb genomic sequences resulted in a 3.8-kb fragment of *dnlg4* genomic DNA (including the first to fifth exons of the *dnlg4* gene) being replaced with the mini-white transgene (Fig. 2*A*). Using a PCR screen, we obtained two *dnlg4* KO lines, *dnlg4^KO7^* and *dnlg4^KO10^*, which were homozygous viable and fertile with no detectable abnormalities in body size or morphology. To verify that both knock-out lines were *dnlg4*-null mutants, we first performed an RT-PCR assay with *dnlg4* primers to examine the *dnlg4* mRNA in WT and the two mutant lines. The mRNA of *dnlg4* could be detected in the WT line, but it was undetectable in both knock-out lines (Fig. 2*B*). We further carried out Western blot analyses with larval brain extracts from the WT line, two mutant lines, and a DNlg4-overexpressing line driven by the Elav-Gal4 driver, using the anti-DNlg4 antibody. The predicted 140-kDa band of DNlg4 was detected in WT and DNlg4-overexpressing flies (*dnlg4 OE*), but was absent in both mutant lines (Fig. 2*C*). These results demonstrated that both *dnlg4^KO7^* and *dnlg4^KO10^* were indeed null mutants. In all subsequent experiments, we used *dnlg4^KO10^* as the *dnlg4* mutant.

To elucidate a possible role for DNlg4 in neuromuscular synaptic development, we first examined the NMJ morphology of *dnlg4* mutants. We co-labeled the NMJs with an anti-HRP antibody, which labeled the neuronal membranes, and an anti-DLG antibody, which labeled type I boutons, and carried out an overall morphological analysis for type Ib synaptic boutons of NMJs in muscle 4 (NMJ4) of abdominal segment 2/3. Compared with WT controls (Fig. 2*D*), the overall morphology of NMJs in *dnlg4* mutants appeared normal with a stereotypical “beads-on-a-chain” pattern. However, *dnlg4* mutants showed fewer and shortened axon branches with obviously reduced numbers of boutons, whereas bouton size significantly increased (Fig. 2, *E* and *G*). To eliminate the possibility that these phenotypes were caused by a background mutation on the third chromosome, we also examined the NMJ morphology of *dnlg4* hemizygous mutants *dnlg4*/Df(3R)ED6027 and found that they also exhibited identical morphological defects at NMJs (Fig. 2, *F* and *G*). The phenotype of this synaptic undergrowth was highly penetrant, and we also observed similar morphological defects of NMJs in muscle 6/7 (NMJ6/7) (Fig. 2, *D*′–*F*′ and *H*).

In addition, *in vitro* studies have indicated that Nrxs/Nlgs are required to trigger synaptic differentiation by recruiting presynaptic vesicles and postsynaptic scaffolding proteins and receptors (35, 40, 41, 50). We therefore assessed whether loss of *dnlg4* induced impaired synaptic differentiation. Using immunostaining with different antibodies, we examined the distribution and levels of some synaptic proteins at NMJs, such as the active zone protein BRP, the vesicle-associated protein cysteine string protein (CSP), SYT, the postsynaptic scaffold protein DLG, and the glutamate receptors, including GluRIIA and GluRIIB. However, no statistical alteration of these molecules in *dnlg4* mutants was observed (supplemental Fig. S3).

To further evaluate the role of DNlg4 in synaptic architecture and differentiation, we carried out ultrastructural analyses of larval NMJs in *dnlg4* mutants by electron microscopy. The synaptic ultrastructure of the *dnlg4* mutants appeared grossly normal. We did not observe significant differences in the morphology and the number of presynaptic vesicles, the active zone (AZ), or the T-bar in a single bouton between the WT and *dnlg4* mutant lines (Fig. 2, *I* and *J*). However, the bouton area per active zone was statistically increased in *dnlg4* mutants, and the single postsynaptic density (PSD) length had an apparent increase in *dnlg4* mutants, although the total PSD length per perimeter was statistically normal compared with the WT controls. A striking defect in the *dnlg4* mutants appeared in the active zone structure: the presynaptic membrane within the active zone was detached from the opposite postsynaptic membrane at several points and displayed invaginations or ruffles, and the typical electron density of presynaptic membranes and the synaptic cleft in these sites were lost (Fig. 2, *I*′, *J*′, and *K*). However this phenotype rarely appeared in the WT flies. In addition, we also observed a postsynaptic defect in the *dnlg4* mutants. The area of subsynaptic reticulum (SSR) per bouton was significantly decreased in the *dnlg4* mutants (Fig. 2, *I–K*).

Taken together, these results revealed an indispensable role of DNlg4 during larval NMJ development, especially for proper proliferation of synaptic boutons and the correct synaptic architecture of NMJs.

### DNlg4 presynaptically promotes the proliferation of synaptic boutons of NMJs

**Figure 3.**
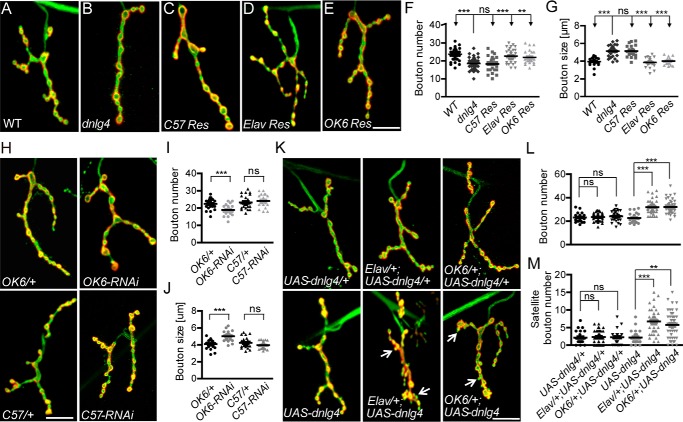
**Presynaptic DNlg4 positively regulates NMJ growth.** *A–E*, representative NMJ4 morphology in the WT line (*A*), *dnlg4* mutants (*B*), a muscle-specific rescue line using C57-Gal4 (*C57 Re*s) (*C*), a pan-neuronal rescue line using Elav-Gal4 (*Elav Res*) (*D*), and a motoneuron-specific rescue line using OK6-Gal4 (*OK6 Res*) (*E*), co-staining with anti-HRP antibody and anti-DLG antibody. *F* and *G*, quantification of the NMJ4 bouton number and bouton size in the genotypes indicated in *A–E. H*, representative NMJ4 morphology in an *OK6*/+ line, a motoneuron-specific RNAi line (*OK6-RNAi*), a *C57*/+ line, and a muscle-specific RNAi line (*C57-RNAi*). *I* and *J*, quantification of the NMJ4 bouton number and bouton size in the genotypes indicated in *H. K*, representative NMJ4 morphology in flies overexpressing one and two copies of the *dnlg4* transgene driven by the Elav-Gal4 driver and OK6-Gal4 driver. The satellite boutons are indicated by *arrows. L* and *M*, quantification of the total bouton number and the satellite bouton number at NMJ4 in genotypes indicated in *K*. For all comparisons, the Mann–Whitney test was used. ***, *p* < 0.001; **, *p* < 0.01; *ns*, no significance. *Scale bar* in all *images*, 20 μm.

To clarify whether the developmental defects of NMJ in *dnlg4* mutants were due to the loss of DNlg4 in the presynapse or postsynapse of NMJs, we carried out rescue experiments by expressing *UAS-dnlg4* with the tissue-specific Gal4 driver in *dnlg4* mutants. Because Nlgs are thought to act at the postsynaptic areas as the postsynaptic ligands of Nrxs in mammals (21, 23, 51), we first expressed *UAS-dnlg4* in muscles using a muscle-specific C57-Gal4 driver, but we did not observe any rescue effect (Fig. 3, *A–C*, *F*, and *G*). Conversely, when exogenous DNlg4 was expressed in neurons using the Elav-Gal4 driver, the morphological defects were completely rescued (Fig. 3, *D*, *F*, and *G*). To verify that these morphological defects in NMJs were exclusively due to the loss of DNlg4 in motoneurons, we performed rescue experiments specifically in motoneurons with the OK6-Gal4 driver, and a complete rescue effect was observed (Fig. 3, *E–G*). Thus, the developmental defects of NMJ in *dnlg4* may derive from the loss of DNlg4 in presynaptic motoneurons instead of in postsynaptic muscle cells.

To further confirm this independently, we conducted RNAi experiments using UAS-*dnlg4*-RNAi constructs driven by motoneuron-specific and muscle-specific Gal4 drivers to knock down the protein level of DNlg4. Compared with controls, knockdown of DNlg4 in motoneurons with the OK6-Gal4 driver led to decreased bouton number and increased bouton size similarly to the *dnlg4* mutants. However, knockdown of DNlg4 in muscles with the C57-Gal4 driver did not cause these morphological defects (Fig. 3, *H–J*).

Because loss of DNlg4 in the presynapse caused an undergrowth of NMJs, we hypothesized that overexpressing DNlg4 presynaptically would lead to NMJ overgrowth. To test this hypothesis, one copy of *UAS-dnlg4* was expressed in the WT flies with the Elav-Gal4 driver and the OK6-Gal4 driver, respectively. Compared with controls, we did not observe a significant increase in the number of synaptic boutons. However, when we expressed DNlg4 at high levels by driving two copies of *UAS-dnlg4* with these two Gal4 drivers, we observed a significant increase in bouton number, accompanied with many satellite boutons (Fig. 3*K*). We defined satellite boutons as small boutons emanating from the main branch or from primary boutons (2, 52). Quantification data showed that the mean total bouton number in *dnlg4* mutants increased by about 40% (Fig. 3*L*), and the mean number of satellite boutons was about 3-fold higher than that in WT controls (Fig. 3*M*), suggesting that the synaptic overgrowth was specifically attributable to high levels of DNlg4 in motoneurons. We also expressed one or two copies of *UAS-dnlg4* in muscle cells with the C57-Gal4 driver, but no increase in the number of synaptic boutons was observed (supplemental Fig. S4). These results indicated that overexpression of DNlg4 presynaptically was sufficient to promote the proliferation of synaptic boutons at NMJs and further demonstrated that DNlg4 functions presynaptically, but not postsynaptically, in regulating NMJ growth.

### DNlg4 has a genetic interaction with components of the BMP pathway

BMP signaling constitutes the primary positive retrograde growth signal in NMJ development. Down-regulation of BMP signaling leads to a smaller NMJ size with reduced bouton number (9–12), whereas up-regulation of BMP signaling induces NMJ overgrowth with more boutons, including satellite boutons (2, 12, 13). These phenotypes were similar to what we observed in *dnlg4* mutants and in *dnlg4*-overexpressing flies. We therefore hypothesized that the morphological defects in NMJs induced by loss or gain of DNlg4 were related to BMP signaling.

**Figure 4.**
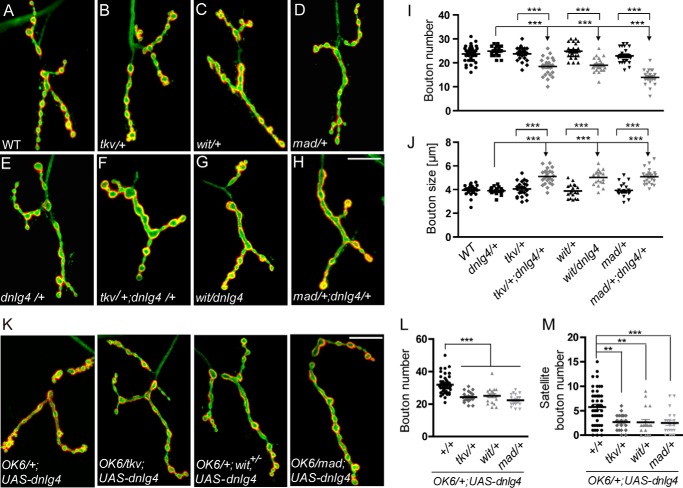
**DNlg4 genetically interacts with components of the BMP pathway in regulating NMJ growth.** *A–H*, representative NMJ4 morphology in the following genotypes: WT (*A*), *tkv*/+ (*B*), *wit*/+ (*C*), *mad*/+ (*D*), *dnlg4*/+ (*E*), *tkv*/+;*dnlg4*/+ (*F*), *wit/dnlg4* (*G*), and *mad*/+;*dnlg4*/+ (*H*). *I* and *J*, quantification of bouton number and bouton size in NMJ4 from genotypes indicated in *A–H. K*, representative NMJ4 morphology in *OK6*/+;*UAS-dnlg4* line, *OK6/tkv*;*UAS-dnlg4* line, *OK6*/+;*wit*^+/−^,*UAS-dnlg4* line, and *OK6/mad*;*UAS-dnlg4* line. *L* and *M*, quantification of the total bouton number and the satellite bouton number at NMJ4s from the genotypes indicated in *K*. For all comparisons, the Mann–Whitney test was used. ***, *p* < 0.001; **, *p* < 0.01. *Scale bar*, 20 μm.

To test this hypothesis, genetic interactions between *dnlg4* and components of the BMP pathway were examined. Larvae heterozygous for the *dnlg4* mutation had a normal bouton number and bouton size at NMJs compared with WT controls. Similar to *dnlg4* mutant heterozygotes, the number and the size of NMJ boutons in heterozygous mutants for the BMP type I receptor, *tkv*, the type II receptor, *wit*, and the BMP effector, *mad*, were all similar to that found in WT controls (Fig. 4, *A–E*, *I*, and *J*). However, the transheterozygotes for *tkv*/+;*dnlg4*/+, *wit/dnlg4* and *mad*/+;*dnlg4*/+ had significantly decreased bouton numbers and apparent increased bouton size compared with each heterozygous line (Fig. 4, *F–J*). In addition, overexpression of two copies of *UAS-dnlg4* presynaptically in the WT background induced obvious NMJ overgrowth with a pronounced increase in total bouton number and satellite bouton number. However, the overgrowth of NMJs in this line was suppressed by mutating one copy of *tkv*, *wit*, or *mad* (Fig. 4, *K–M*). These results indicated that *dnlg4* had a dosage-sensitive genetic interaction with the components of the BMP pathway and that the NMJ growth promoted by DNlg4 overexpression required BMP signaling.

### DNlg4 regulates NMJ growth by positively modulating BMP signaling

**Figure 5.**
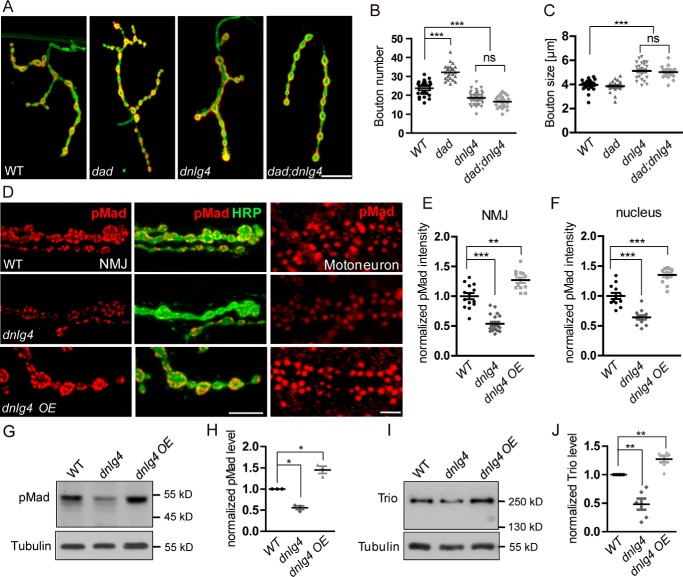
**DNlg4 regulates NMJ growth by modulating BMP signaling.** *A*, representative NMJ4 morphology in the WT, *dad*, *dnlg4*, and *dad*;*dnlg4* lines. *B* and *C*, quantification of bouton number and bouton size in the genotypes indicated in *A*. The Mann–Whitney test was used. *D–F*, the level of pMad at the synaptic boutons of NMJs and in the motoneuron nuclei from the WT, *dnlg4* mutant, and *dnlg4*-overexpressing flies driven by the OK6-Gal4 driver (*dnlg4 OE*) (*D*), showing decreased pMad in *dnlg4* mutants and increased pMad in *dnlg4 OE* flies. The fluorescent intensities of pMad were quantified in *E* and *F*. The Mann–Whitney test was used. *G* and *H*, Western blot analyses of pMad using larval brain extracts from the WT, *dnlg4*, and *dnlg4 OE* lines. β-Tubulin was used as the loading control. Quantification of pMad on Western blots is shown in *H*. The paired *t* test was used. *I* and *J*, Western blot analyses for Trio using larval brain extracts from the WT, *dnlg4*, and *dnlg4 OE* lines, showing that the expression of Trio is decreased in the *dnlg4* mutants and increased in the *dnlg4 OE* flies. β-Tubulin was used as the loading control. Quantification of Trio on Western blots is shown in *J*. The paired *t* test was used. ***, *p* < 0.001; **, *p* < 0.01; *, *p* < 0.5 for all comparisons. *Scale bar*, 20 μm (*A*); 10 μm (*D*, for NMJ), and 20 μm (*D*, for motoneuron).

Because DNlg4 genetically interacted with components of the BMP pathway, we hypothesized that DNlg4 regulated the synaptic growth of NMJs by modulating BMP signaling. An analysis of the genetic interactions between *dnlg4* and daughters against DPP (*dad*) supported this hypothesis. Dad (daughters against DPP) acts as an inhibitor of the BMP signaling pathway by competitively binding to Tkv with Mad. Mutation of *dad* leads to an increase in BMP signaling and promotes NMJ overgrowth (2, 53). Consistent with previous reports, we also observed a significant increase in bouton number in *dad* mutants. However, in *dad*;*dnlg4* double mutants, the NMJ overgrowth was completely reversed, and the bouton number was decreased to the same level as seen in the *dnlg4* mutants, accompanied with increased bouton size (Fig. 5, *A–C*). A reasonable explanation for this phenomenon is that loss of DNlg4 decreases the total BMP signaling level in *dad*;*dnlg4* mutants.

In the BMP pathway, phosphorylated Mad (pMad) serves as a molecular readout of BMP signaling, and the level of pMad is positively correlated with larval NMJ growth in *Drosophila* (2). We therefore measured the levels of pMad in NMJs and in motoneuron nuclei of *dnlg4* mutants and *dnlg4-*overexpressing flies using immunostaining with anti-pMad antibodies. The pMad level in NMJs was decreased by 47% in *dnlg4* mutants compared with WT control. Conversely, overexpressing DNlg4 in a WT background driven by the OK6-Gal4 driver led to an ∼30% increase in pMad levels. A similar result was observed in motoneuron nuclei (Fig. 5, *D–F*). The Western blot analyses of extracts from larval brains with anti-pMad antibodies confirmed this result (Fig. 5, *G* and *H*). In addition, we also examined the expression of *trio*, one of the BMP signaling target genes, that is required for normal NMJ growth (3). Western blot analyses of larval brain homogenates with anti-Trio antibodies showed that Trio was significantly decreased in the *dnlg4* mutants but increased in the *dnlg4*-overexpressing flies (Fig. 5, *I* and *J*). Taken together, these results demonstrated that DNlg4 positively regulated BMP signaling, which, in turn, affected the synaptic growth of NMJs.

### DNlg4 physically interacts with Tkv and regulates the Tkv protein level

**Figure 6.**
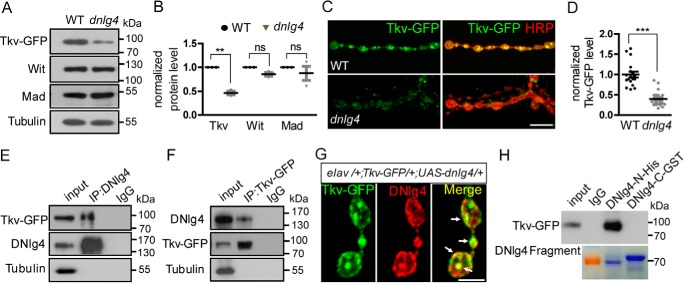
**DNlg4 physically interacts with Tkv and regulates Tkv protein levels in presynapse.** *A* and *B*, Western blot analyses of total larval brain extracts from the control (*WT*;*Elav-Gal4*>*UAS-Tkv-GFP*) and mutants (*dnlg4*;*Elav-Gal4*>*UAS-Tkv-GFP*) probed with anti-GFP, anti-Wit, and anti-Mad antibodies, showing a decrease of Tkv-GFP proteins in *dnlg4* mutants (quantified in *B*). β-Tubulin was used as the loading control. The paired *t* test was used. *C* and *D*, synaptic boutons of NMJs from control (*WT*;*Elav-Gal4*>*UAS-Tkv-GFP*) and *dnlg4* mutants (*dnlg4*;*Elav-Gal4*>*UAS-Tkv-GFP*) labeled with anti-GFP (*green*) and anti-HRP (*red*) (*C*), showing that the Tkv-GFP in the synaptic boutons was decreased in the *dnlg4* mutants (quantified in *D*). The Mann–Whitney test was used. *E* and *F*, co- immunoprecipitation (*IP*) analysis with anti-DNlg4 and anti-GFP using fly head homogenates from flies co-overexpressing DNlg4 and Tkv-GFP, driven by the Elav-Gal4 driver, showed specific co-immunoprecipitation of DNlg4 and Tkv-GFP. The monoclonal antibodies against IgG were used as the negative control. *G*, synaptic boutons of NMJ4 from flies co-overexpressing DNlg4 and Tkv-GFP with the Elav-Gal4 driver, labeled with anti-GFP (*green*) and anti-DNlg4 (*red*), showing co-localization of Tkv-GFP and DNlg4 at presynaptic membranes and in endosomes. *H*, pulldown assays using fly head homogenates from Tkv-GFP–overexpressing flies with recombinant protein DNlg4-N-His and DNlg4-C-GST showed that Tkv-GFP was pulled down by the N-terminal region of DNlg4. The monoclonal antibodies against IgG were used as the negative control. The recombinant proteins were stained with Coomassie Blue (shown as *blue bands*; the *red band* is the 70-kDa protein marker). ***, *p* < 0.001; **, *p* < 0.01; *ns*, no significance for all comparisons. *Scale bars*, 10 μm (*C*) and 5 μm (*G*).

A key question is the mechanism underlying the down-regulation of pMad in *dnlg4* mutants. A previous study reported that mutations of *wit*, *tkv*, or *mad* separately led to undergrowth of NMJs, and this was due to the down-regulation of pMad (12). We therefore first examined the levels of these three proteins in *dnlg4* mutants by Western blotting of larval brain homogenates. Because there was no available antibody against Tkv, an anti-GFP antibody was utilized to detect the ectopically expressed Tkv-GFP driven by the Elav-Gal4 driver in the *dnlg4* mutants and WT controls. Compared with the controls, the protein levels of Wit and Mad were not changed, but the Tkv-GFP in the *dnlg4* mutant background was significantly reduced (Fig. 6, *A* and *B*). We next co-stained larval body wall muscles with anti-GFP and anti-HRP antibodies to determine whether the Tkv-GFP protein in presynaptic areas of NMJs is altered in the *dnlg4* mutants. Compared with the controls, the Tkv-GFP at NMJs was decreased by about 60% in the *dnlg4* mutants (Fig. 6, *C* and *D*, and supplemental Fig. S5*A*). Consistently, a similar reduction of Tkv-GFP in VNC in the *dnlg4* mutants was also observed (supplemental Fig. S5, *B* and *C*). These results suggested the specific regulation of Tkv by DNlg4.

To determine whether the reduction of Tkv proteins in *dnlg4* mutants was due to the down-regulation of *tkv* transcripts, we carried out RT-PCR analyses of the mRNA extracted from larval brain homogenates using *tkv-*specific primers. The mRNA level of *tkv* in the *dnlg4* mutants was not altered (supplemental Fig. S6), suggesting a posttranscriptional regulation of Tkv by DNlg4. A trafficking defect of Tkv from the cell body to the axon terminal could be excluded, because no excess accumulation of Tkv-GFP in the soma of VNC motoneurons was observed (supplemental Fig. S5*B*). Thus, we speculated that DNlg4 probably regulated the Tkv protein level by affecting its recruitment and/or stability in the presynaptic regions. If correct, DNlg4 should have a physical interaction with Tkv. To confirm this, we carried out immunoprecipitation studies with the anti-DNlg4 antibodies using adult head homogenates from flies that co-overexpressed DNlg4 and Tkv-GFP driven by the Elav-Gal4 driver. The subsequent Western blotting of the immunoprecipitants with the anti-GFP antibody showed co-immunoprecipitation of Tkv and DNlg4 (Fig. 6*E*). A reverse co-immunoprecipitation assay was performed by using anti-GFP antibodies to precipitate Tkv-GFP, and the DNlg4 could also be detected by subsequent Western blotting of the same homogenates (Fig. 6*F*). This result indicated that DNlg4 physically interacted with Tkv *in vivo*. As further support, we also observed co-localization of DNlg4 and Tkv-GFP in the presynaptic membrane and endosomes at NMJs (Fig. 6*G*). To further verify which domain in DNlg4 protein interacted with Tkv, we expressed two fused proteins, including a DNlg4 N-terminal fragment (amino acids 1–675, containing the acetylcholinesterase-like domain) fused with a His tag and a C-terminal fragment (amino acids 848–1281) fused with a GST tag in *Escherichia coli* BL21 cells. We used these two fused proteins to precipitate Tkv-GFP from the adult head homogenates that were from *tkv-gfp* overexpressing flies and driven by the Elav-Gal4 driver. The Tkv-GFP could be precipitated with the N-terminal, instead of C-terminal, region of DNlg4 protein (Fig. 6*H*), indicating that the interaction between DNlg4 and Tkv was dependent on the acetylcholinesterase-like domain of DNlg4.

### The dnlg4 mutants display decreased neurotransmission and reduced locomotor activity

**Figure 7.**
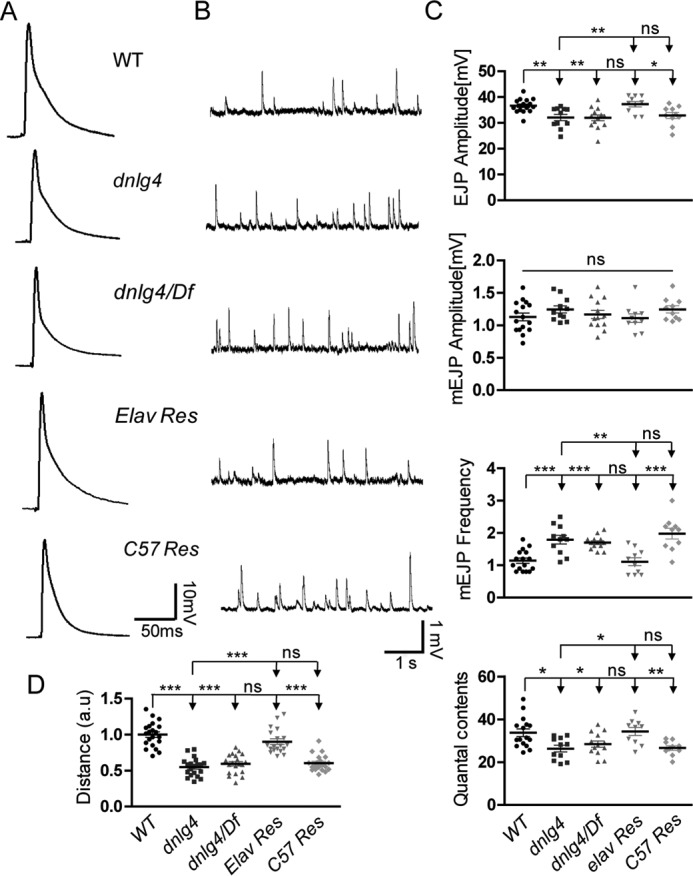
**Impaired synaptic transmission and reduced locomotor activity in the *dnlg4* mutants.** *A*, representative traces of EJPs in the indicated genotypes, showing that the amplitudes of EJPs were decreased in both *dnlg4* mutants and *dnlg4/Df* heterozygous mutants and were restored when expressing exogenous DNlg4 in neurons. *B*, representative traces of mEJPs in the indicated genotypes, showing increased frequency of the mEJPs in both the *dnlg4* mutant and *dnlg4/Df* flies. This defect was rescued by expression of exogenous DNlg4 in neurons. *C*, quantification of the EJP and mEJP amplitudes, mEJP frequency, and quantal contents in the indicated genotypes. *D*, quantification of a 3-min crawling distance of the third-instar larvae showing a reduced locomotor activity in both the *dnlg4* mutants and *dnlg4*/*Df* flies and restoration by neural expression of exogenous DNlg4 (*a.u.*, artificial unit). For all comparisons, the Mann–Whitney test was used. ***, *p* < 0.001; **, *p* < 0.01; *, *p* < 0.05.

Generally, abnormal development of NMJs leads to functional defects. To assess the functional consequences of impaired synapse development in NMJs in *dnlg4* mutants, an electrophysiological analysis of NMJs was carried out using intracellular recordings at 0.5 mm Ca^2+^. The evoked excitatory junction potentials (EJPs), which reflect the evoked transmitter release, and the miniature excitatory junction potentials (mEJPs), which reflect the spontaneous transmitter release, were recorded. The amplitudes of EJPs displayed a small but significant decrease in both the homozygous *dnlg4* mutants and *dnlg4*/Df lines compared with the WT controls (Fig. 7, *A* and *C*). The amplitude of mEJPs in both mutants were not altered; however, the frequency of mEJPs was dramatically increased by about 50–60% (Fig. 7, *B* and *C*). We also analyzed the quantal content, a measure of synaptic transmission efficacy that is calculated by dividing the EJP amplitude by the mEJP amplitude, and found that the quantal contents in both mutants were slightly reduced compared with WT controls (Fig. 7*C*), indicating decreased efficiency of transmitter release at NMJs. Consistent with the morphology, all of the functional defects of NMJs in the *dnlg4* mutants were completely rescued when expressing a *dnlg4* transgene in neurons with the Elav-Gal4 driver, but they were not rescued when transgenic *dnlg4* was expressed in muscles driven by the C57-Gal4 driver (Fig. 7, *A–C*).

An important question, therefore, was whether the functional defects of NMJs would cause behavioral impairments. We performed larval locomotor activity detection in *dnlg4* mutants and WT flies. Compared with WT flies, both *dnlg4* and *dnlg4*/Df mutant flies showed reduced locomotor activities. This behavioral defect was also rescued by expressing a *dnlg4* transgene in neurons, but not in muscles (Fig. 7*D*). In summary, our results showed that loss of DNlg4 led to synaptic transmission defects at NMJs along with reduced larval locomotor activity, and all of the defects were derived from loss of DNlg4 presynaptically.

## Discussion

Most studies of Nlgs/Nrxs have focused on their functions in synapse formation and/or maturation at the single-synapse level. However, recent studies from loss of function of *Drosophila* Nlg1–3 have suggested an important role of DNlgs in neuromuscular synapse development (43–46). Here, we dissected the role of DNlg4 in synapse development and found that DNlg4 presynaptically regulated neuromuscular synaptic growth by modulating BMP signaling.

### The role of DNlg4 in synaptic architecture, synapse development, and function

Sequence analyses showed that there are four *nlg* genes in the *Drosophila* genome, and all four DNlgs share significant amino acid sequence homology and protein structures with vertebrate Nlgs (44). DNlg1 and DNlg2 have a positive effect on synaptic growth of NMJs, as indicated by an obvious reduction in synaptic boutons in the *dnlg1* and *dnlg2* mutants (43–45). Conversely, loss of DNlg3 led to increased numbers of synaptic boutons at NMJs (46). In the present study, a *dnlg4* null mutant was generated by gene targeting, and this mutant exhibited significant defects in NMJ morphology, including fewer synaptic boutons and increased bouton size. However, neuronal overexpression of two copies of the *dnlg4* transgene induced a pronounced increase in bouton number. These results demonstrated a positive role of DNlg4 in regulating synapse development. Interestingly, neuronal overexpression of one copy of *dnlg4* in the WT background did not induce an increase in bouton number, but it did when expressed in a *dnlg4* mutant background. These results suggested a homeostatic adjustment during synapse development in *Drosophila*, which could somewhat counteract the effect caused by increased DNlg4. As a result, a moderate increase of DNlg4 in WT flies did not lead to increased synaptic growth of NMJs.

In *Drosophila*, loss of DNlgs in the *dnlg1–3* mutants also induced synaptic differentiation defects that were characterized by decreased protein levels or impaired distribution of glutamate receptors and other postsynaptic proteins at NMJs (43, 44, 46). In contrast to other *dnlg* mutants, we did not detect statistical alteration in the distribution or protein level of glutamate receptors at NMJs in *dnlg4* mutants. In addition, the distribution and protein level of some presynaptic proteins, such as BRP, CSP, and SYT, were normal in the *dnlg4* mutants. However, the ultrastructural analyses of the NMJs showed that there were still some defects in synaptic ultrastructure in the *dnlg4* mutants, including the increased bouton area per active zone, longer single PSD, and reduced postsynaptic SSR regions. One striking ultrastructural defect in the *dnlg4* mutants was the partial detachment of presynaptic membranes from postsynaptic membranes within the active zone, which was rarely observed in WT flies, suggesting the adhesion function of DNlg4 during synaptogenesis. It was interesting that this defect also appeared in *dnlg1* mutants and *dnrx* mutants (43, 54), suggesting that the DNlg4 might affect the synaptic architecture by a mechanism similar to that of DNlg1 and DNrx.

Functionally, *dnlg4* mutants showed a mild impairment in transmitter release at NMJs, as characterized by slightly reduced evoked EJP amplitude and quantal contents. This phenotype was consistent with the morphological impairments and the ultrastructural defects of NMJs, suggesting a probable reduction in the number of total synapses or functional synapses at NMJs. The amplitude of mEJP was not changed in the *dnlg4* mutants, which was consistent with the normal protein levels of postsynaptic glutamate receptors. Interestingly, the frequency of mEJPs in the *dnlg4* mutants was dramatically increased, indicating that DNlg4 affects spontaneous transmitter release. The detailed mechanism underlying this should be addressed in future work.

### DNlg4 functions presynaptically in synaptic growth and transmitter release at NMJs

In mammals, Nlgs are generally considered to function as postsynaptic adhesion molecules and to help form trans-synaptic complexes with presynaptic Nrxs (23, 50, 51, 55). In *Drosophila*, DNlg1 and DNlg3 are reported to be located in postsynaptic membranes (43, 46). However, there are some exceptions to the postsynaptic localization of Nlgs. For example, an Nlg in *Caenorhabditis elegans* is reported to be present in both presynaptic and postsynaptic regions (56). DNlg2 is also required both presynaptically and postsynaptically for regulating neuromuscular synaptic growth (45). These studies support a more complex mechanism of Nlgs in synapse modulation and function. Our data add to this complexity by suggesting a presynaptic role of DNlg4 in larval neuromuscular synaptic growth and synaptic functions.

First, in the VNC of third-instar larvae, DNlg4 was concentrated in the neuropil region where the synapses aggregated. In NMJs, although endogenous DNlg4 was not detected by our anti-DNlg4 antibodies, the exogenous DNlg4 that was expressed in motoneurons was located in type I synaptic boutons of NMJs, suggesting a reasonable presynaptic location of DNlg4 at NMJs. In another assay, DNlg4 was expressed using a DNlg4-Gal4 driver to mimic the endogenous expression pattern of the *dnlg4* gene. The DNlg4 promoted by DNlg4-Gal4 was distributed in type I boutons of NMJs and was located in presynaptic areas of boutons, which also supported the presynaptic location of endogenous DNlg4 at NMJs, although a simultaneous postsynaptic localization of DNlg4 at NMJs could not be excluded. Second, knockdown of DNlg4 presynaptically led to morphological defects in NMJs similar to those observed in the *dnlg4* mutants, indicated by decreased bouton numbers and increased bouton size. However, knockdown of DNlg4 postsynaptically did not cause the same phenotypes. These morphological defects of NMJs in the *dnlg4* mutants could be completely rescued when DNlg4 was expressed in presynaptic neurons, but not when it was expressed in postsynaptic muscles. Third, *dnlg4* mutants had a significant increase in spontaneous transmitter release frequency, which was usually interpreted as a presynaptic defect (54). In addition, all of the functional defects in the *dnlg4* mutants, including decreased amplitude of EJPs, reduced quantal contents, and increased mEJP frequency, could be rescued by presynaptic expression of DNlg4. These results indicated that presynaptic DNlg4 was essential for proper proliferation and function of synapses at NMJs. Finally, the number of synaptic boutons at NMJs was significantly increased when two copies of *UAS-dnlg4* were overexpressed in presynaptic neurons, whereas this phenomenon was not observed when two copies of *UAS-dnlg4* were overexpressed in muscles, suggesting that presynaptic DNlg4 alone was sufficient to promote synaptic growth. Altogether, these data provided convincing evidence that DNlg4 functions as a presynaptic molecule in regulating synaptic growth and transmitter release at NMJs.

### DNlg4 positively regulates BMP signaling through Tkv

The BMPs are major retrograde trans-synaptic signals that affect presynaptic growth and neurotransmission both in the CNS and at NMJs (57–59). In this study, we have presented immunohistochemical and genetic data showing that DNlg4 regulates NMJ growth via the BMP signaling pathway.

First, the *dnlg4* mutants shared similar phenotypes with the components of the BMP signaling pathway in synapse development, synapse architecture, and synapse functions of NMJs, including reduced synaptic bouton number, increased presynaptic membrane ruffles, and decreased synaptic transmission efficacy (9, 12). Second, several genetic crosses followed by neuromuscular bouton number analyses showed a definite dosage-sensitive genetic interaction between *dnlg4* and the components of the BMP signaling pathway, such as *tkv*, *wit*, *mad*, and *dad*, suggesting that DNlg4 was required for BMP signaling in regulating NMJ growth. Third, both immunohistochemical and Western blot analyses showed that the pMad, which serves as an indicator of BMP signaling, was decreased in both the synaptic boutons of NMJs and motoneuronal nuclei in the *dnlg4* mutants, whereas it was increased in *dnlg4*-overexpressing flies. Finally, the expression of the BMP signaling target gene *trio*, was significantly reduced in the *dnlg4* mutants, but it was increased in *dnlg4*-overexpressing flies. Together, these results supported our hypothesis that DNlg4 promoted synaptic growth by positively regulating BMP signaling.

The critical question to be addressed, therefore, is the mechanism underlying this regulation. Through Western blot analyses of larval brain homogenates, we found that the protein level of Tkv (as assessed by ectopically expressed Tkv-GFP) in the *dnlg4* mutants was significantly decreased, whereas Wit and Mad, the other two components of the BMP pathway, were not altered. The immunohistochemical assay also showed that the Tkv protein in the *dnlg4* mutants was reduced in both the VNC and the synaptic boutons of NMJs. These data indicated the specific positive regulation of Tkv by DNlg4. Previous studies reported that *tkv* mutants had decreased synaptic bouton number, increased presynaptic membrane ruffles, reduced amplitude of EJP, and unchanged mEJP amplitude (12, 15). In addition, neuronal overexpression of one copy of transgenic *tkv* did not induce NMJ overgrowth, but it induced such overgrowth when two copies of transgenic *tkv* were expressed in neurons (2). These phenotypes were similar to what we observed in the *dnlg4* mutants and *dnlg4*-overexpressing flies. All of these results strongly demonstrated that DNlg4 regulates BMP signaling by modulating Tkv protein levels.

Because the *dnlg4* mutants had a level of *tkv* mRNA comparable with that of the WT controls, DNlg4 did not affect the transcription of *tkv*. The possibility of Tkv trafficking defects from the cell body to the axon terminal could also be excluded because no retention of Tkv in the soma of motoneurons was observed. Thus, it seems reasonable to speculate that DNlg4 affects the recruitment or stability of Tkvs at the presynapse. In support of this hypothesis, co-localization of Tkv and DNlg4 at presynaptic regions of NMJs was observed by immunostaining. As further confirmation, we showed that Tkv could be co-immunoprecipitated with an antibody against DNlg4, and a reverse co-immunoprecipitation assay using anti-GFP antibodies also resulted in the precipitation of DNlg4 by Tkv-GFP, suggesting a physical interaction between DNlg4 and Tkv *in vivo*. Furthermore, using pull-down assay, we proved that the acetylcholinesterase-like domain of the N terminus was essential for DNlg4 interacting with Tkv.

If the recruitment of Tkv to the presynaptic membrane entirely depends on DNlg4, we would not detect the existence of Tkv in the presynaptic membrane, and accumulation of Tkv proteins in the presynaptic areas of NMJs would probably be observed in the *dnlg4* mutants. However, we detected minor Tkv protein level at the presynaptic membrane, and no accumulation of Tkv was observed at NMJs in the *dnlg4* mutants. Thus, a more reasonable hypothesis is that DNlg4 affected the stability of Tkv at the presynaptic membranes. The protein level of Tkv in the presynapses of NMJs was reported to be regulated by several pathways, including direct proteasomal degradation, ubiquitin-mediated degradation, and endocytosis (2, 15, 60, 61). The simplest model is that the DNlg4 might stabilize Tkv via inhibiting its degradation. Several protein kinases, such as the ribosomal protein S6 kinase-like protein (S6KL) and the serine/threonine kinase Fused, have been reported to interact physically with Tkv *in vitro* and to facilitate its proteasomal degradation (15, 61). In particular, S6KL has been demonstrated to degrade Tkv at NMJs. DNlg4 might stabilize Tkv by inhibiting these protein kinases, but the detailed mechanism of such activity still needs to be addressed.

In summary, this study demonstrated that DNlg4 positively regulated neuromuscular synaptic growth by modulating BMP signaling through the maintenance of Tkv protein levels in the presynapse of NMJs. To accomplish this function, DNlg4 acted as a presynaptic molecule instead of a postsynaptic molecule. Our study further suggested a relationship between Nlgs and BMP signaling and provided a new understanding of the exact role of Nlgs during synapse formation and development.

## Experimental procedures

### Drosophila stocks

The *w^1118^* strain was used as the WT control in this study. The *UAS-Tkv-GFP* transgenic flies were supplied by M. Gonzalez-Gaitan (University of Geneva). The *OK6-Gal4* line was from M. O'Connor (University of Minnesota, Saint Paul, MN), and the *C57-Gal4* line was from V. Budnik (University of Massachusetts School of Medicine, Worcester, MA). *UAS-dnlg4-RNAi* flies were purchased from the Vienna Drosophila RNAi Center (catalog no. V6791). *UAS-dnlg4* transgenic flies, *UAS-dnlg4-EGFP* transgenic flies and *DNlg4-Gal4* flies were generated in our laboratory. The remaining strains, Df(3R)ED6027 (a deficiency that removes *dnlg4*), *Mhc-Gal4*, *Elav-Gal4*, *syt-gfp*, *dscam-gfp*, *witA12*, *tkv7*, *mad8-2*, and *dadj1E4*, were obtained from the Bloomington Stock Center. All stocks were cultured in standard medium at 25 °C.

### Generation of transgenic flies and DNlg4-Gal4 flies

*UAS-dnlg4* transgenic flies were generated by embryo injection into *w^1118^* with a recombinant pUAST vector, which contains a full-length *dnlg4* cDNA subcloned from GH07829 cDNA (GenBank^TM^ accession number BT050584). Genomic PCR with primers (5′-ATGGGGGAAAGTCAGCTGCT-3′/5′-TCAGACGCGCATCTCGTCCA-3′) was performed to screen the transgenic line. Western blotting and immunostaining with anti-DNlg4 antibodies were performed to identify this transgenic line. To generate *UAS-dnlg4-EGFP* transgenic flies, full-length DNlg4-GFP was generated by inserting GFP between amino acids Leu^859^ and Gln^860^ (see supplemental Text S1). To generate *DNlg4-Gal4* flies, a 6-kb genomic sequence of *dnlg4* flanking the transcription start site of the *dnlg4* gene (containing an upstream 5-kb and downstream 1-kb DNA sequence) was cloned to pPT-Gal4 vector, followed by embryo injection into *w^1118^*.

### Generation of the dnlg4 null mutant

Ends-out technology (49) was used to generate *dnlg4* knock-out flies. In brief, two homologous fragments (upstream arm, −3954 to −947; downstream arm, 2872–6253) of the *dnlg4* locus were amplified from *w^1118^* genomic DNA with two pairs of primers (5′-GGTCGTACGAGCTGATGAGCAAGAATATC-3′/5′-GAAGGCGCGCCCATGTATGTATCTGCTTGGT-3′; 5′-TACGGTACCACAGTTGGCGGATGAAAGAA-3′/5′-TATGCGGCCGCCGCCAAAGTAGCCATACAGC-3′) and then were subcloned into the pW25 vector (obtained from the Drosophila Genomics Resource Center). Transgenic flies were generated by injecting this recombinant vector into *w^1118^* embryos. Virgin donor flies bearing the targeting construct on the third chromosome were crossed to *yw*; P(70hsFLP70hsI-S-ceI)/Cyo flies (Bloomington Stock Center catalog no. 6934) and the first-instar progeny larvae were heat-shocked for 60 min at 38 °C to induce homologous recombination. Genomic PCR with primers (5′-TGTGGGCGGTAAATGAGTCC-3′/5′-CGCCAAAGTAGCCATACAGC-3′) was performed to screen the targeted flies. Two independent targeted lines, *dnlg4^KO7^* and *dnlg4^KO10^*, were obtained and further confirmed by RT-PCR with primers (5′-GGACTGCCTGTACCTAAATG-3′/5′-GACGTAGGTGCGGATAATTT-3′) and Western blot analyses with anti-DNlg4 antibodies.

### Generation of antibodies against DNlg4

Polyclonal antibodies against DNlg4 were generated in rabbits. A purified glutathione *S*-transferase fusion fragment (Leu^912^–Thr^1089^) of DNlg4 protein was used as an antigen. The antibody was affinity-purified using a column packed with the same recombinant protein (amino acids 912–1089) fused to Sepharose 4B. A recombinant protein containing the same peptide fragment of DNlg4 fused with a His_6_ tag at the C terminus was used as an antigen to generate monoclonal antibodies in mice.

### Western blot analysis

The procedure for Western blotting was described in a previous report (62). The following primary antibodies were used for Western blot analyses in this study: rabbit anti-DNlg4 (1:1000), rabbit anti-pSmad (1:1000; Cell Signaling, catalog no. 9511S), mouse anti-Trio (1:500; Developmental Studies Hybridoma Bank (DSHB), 9.4A), mouse anti-GFP (1:500; Sigma), mouse anti-Wit (1:1000; DSHB, 23C7), mouse anti-Mad (1:500; Santa Cruz Biotechnology, Inc.), and mouse anti-β-tubulin (1:10,000; Sigma, DM1A). To quantify the target protein levels, positive signals on Western blots from at least three independent repeats were calculated using ImageJ and normalized to the β-tubulin control.

### Immunoprecipitation

Immunoprecipitation experiments were performed as described previously (45). Briefly, adult heads were lysed in radioimmune precipitation assay buffer with protease inhibitor (Roche Applied Science). A total of 25 ml of 50% lysate, 50% protein A-Sepharose plus 3 mg of antibodies against DNlg4 or GFP was incubated at 4 °C overnight. Protein A beads were washed and resuspended in 1× SDS loading buffer (with 100 mm DTT) and analyzed by SDS-PAGE and immunoblotting.

### Immunohistochemistry

The immunostaining procedure of whole-mount embryos and dissected wandering third-instar larvae were described previously (46). In brief, after fixation in 4% paraformaldehyde (for larvae) or in methanol (for embryos) and blocking by 0.5% BSA, the samples were incubated with the primary antibodies at 4 °C overnight. After washes with PBT (PBS + 0.3% Triton X-100), the samples were immunostained by the secondary antibodies at room temperature for 1 h. The following primary antibodies were used in this studies: rabbit anti-DNlg4 (1:100); mouse anti-BRP (1:50; DSHB, nc82); rabbit anti-HRP (1:1000; Jackson ImmunoResearch, West Grove, PA); mouse anti-DLG (1:50; DSHB, 4F3); rabbit anti-pMad (1:500; P. ten Dijke, Leiden University, Leiden, The Netherlands); mouse anti-CSP (1:50; DSHB, 6d6); mouse anti-SYT (1:50; DSHB, 3H2); mouse anti-GluRIIA (1:50; DSHB, 8B4D2), rabbit anti-GluRIIB (1:2000; gift from A. DiAntonio, Washington University, St. Louis, MO); rabbit anti-GFP (1:500; Santa Cruz Biotechnology); and goat anti-HRP (1:500; Jackson ImmunoResearch). The secondary antibodies, including Alexa 488- or 568-conjugated anti-mouse, anti-rabbit, or anti-goat (Invitrogen), were used at 1:500. All images were collected using a Carl Zeiss LSM 510 confocal station and analyzed with ImageJ software (National Institutes of Health).

### Pull-down assay

Recombinant proteins, including DNlg4 N terminus (aa 1–675) fused with His tag and DNlg4 C terminus (aa 848–1281) fused with GST tag were expressed in *E. coli* Bl 21 cells. DNlg4-N-His was incubated with anti-His antibodies (Sigma) and purified by protein G/A. DNlg4-C-GST was purified by glutathione-Sepharose (GE Healthcare). Two purified recombinant proteins were incubated, respectively, with the adult head lysates from Tkv-GFP–overexpressing flies. Tkv was detected by subsequent Western blotting using anti-GFP antibodies. Anti-His antibodies was used as a negative control.

### Electron microscopy

The method of EM for third-instar larval NMJ6/7 was described in previous reports (44, 46). Briefly, third-instar larvae were rapidly dissected in Ca^2+^ solution. The dissected body wall muscles (NMJ6/7; segment A3) were first fixed for 4 h at 4 °C in 2% glutaraldehyde and 2% paraformaldehyde in 0.1 m sodium cacodylate buffer (pH 7.2) and then were fixed at 4% glutaraldehyde overnight. After being washed with washing buffer (0.1 m sodium cacodylate buffer, pH 7.2), the samples were post-fixed for 1.5 h with washing buffer containing 1% osmium tetroxide followed by staining for 1 h on ice with 1% uranyl acetate in distilled water. After being dehydrated at room temperature with increasing ethanol concentrations, the samples were infiltrated in Epon resin and embedded in three successive steps at 30, 45, and 60 °C, each lasting for 24 h. Series of 80–90-nm ultrathin sections were cut with a 35° diamond knife (Diatome) on a Reichert ultracut E ultramicrotome (Leica) and mounted on Formvar-coated grids. The sections were finally stained in uranyl acetate and lead citrate. Micrographs were obtained with a Philips EM 301 transmission electron microscope.

### Electrophysiological recordings

Intracellular recordings for assessing NMJ neurotransmissions were performed as described (44). Briefly, wandering third-instar larvae were dissected in calcium-free HL3.1 saline, and both EJPs and mEJPs were recorded from muscle 6 of abdominal segment A3 in HL3.1 saline containing 0.5 mm Ca^2+^ using intracellular microelectrodes (10–20 megaohms) filled with 3 m KCl. Recordings were performed at 20–22 °C with an Axoclamp 2B amplifier (Molecular Devices) in bridge mode, and the recorded data were processed with pClamp version 10.2 software (Molecular Devices). EJPs were evoked by suprathreshold stimulating pulses at 0.3 Hz using a Grass S48 stimulator (Astro-Grass Inc.). Three EJP responses were collected for each animal, and mEJPs were recorded for a period of 60 s after the EJP recording. Only recordings from cells with resting membrane potentials ranging from −60 to −65 mV were used for analysis.

### Larval locomotion activity detection

The larval locomotor assay was performed as described (46). Individual larvae were placed in the center of 15-cm-diameter transparent dishes with 4% agar covering the bottom. A small amount of purple food colorant was added to the agar gel to obtain a dark purple color. The movement of the larvae was visualized via a standard commercial video camera, and the trajectory over 3 min was tracked by tracker software written in Python. A 3-min trajectory distance was calculated for assessing larval locomotion activity.

### Analysis of synaptic boutons of NMJs

Wandering third-instar larvae were dissected, and the body wall muscles were immunostained with anti-HRP and anti-DLG antibodies. The serial confocal images of NMJs of muscle 4 of abdominal segments A2 and A3 were acquired. The individual synaptic boutons could be identified, and the number of type Ib boutons was counted. Bouton size was quantified as the average diameter of the three largest boutons at each NMJ4 measured with ImageJ software.

### Quantification of fluorescence intensity

For comparisons of fluorescent intensities between genotypes, the samples were processed under identical conditions and were immunostained in the same vials. The confocal images of VNC or NMJ4 in the A2/3 segment of each animal were acquired using the same microscope settings. All assays were repeated at least three times. For each channel, the sum of pixel intensities was recorded by ImageJ software. The ratio of fluorescent intensities of the target protein *versus* the control protein was calculated and normalized to the control. The intensity of HRP and BRP were used as the internal control in the quantification of NMJs and VNC, respectively.

### Quantification of tkv mRNA

Total RNA was extracted from the brains of third-instar larvae, and RT-PCR was performed with *tkv* primers (5′-GAATGTTCAGCCAGACGTCG-3′/5′-GCATAAACACGGACAGGGAG-3′). β-Tubulin mRNA was used as an internal control and was amplified with primers 5′-ATGGACTCTGTGCGATCGGG-3′/5′-TTAGTTCTCGTCGACCTCAG-3′. The positive signals of *tkv* cDNA from three independent repeats were calculated with the ImageJ software and normalized to the control.

### Statistical analysis

Statistical significance was determined using Student's *t* tests for comparisons of two group means. Data are expressed as the means ± S.E. *p* values < 0.05 were considered to be statistically significant.

## Author contributions

W. X. conceived the project; W. X. and X. Z. designed the experiments and wrote the manuscript; X. Z., M. R., G. G., J. Y., and C. H. performed the experiments; H. L. helped in data recoding; and W. X. and X. Z. analyzed the data.

## Supplementary Material

Supplemental Data

## Footnotes

2 The abbreviations used are:

NMJ: neuromuscular junction
Nlg: neuroligin
BMP: bone morphogenetic protein
Gbb: glass bottom boat
Wit: wishful thinking
Tkv: thickvein
Mad: mothers against decapentaplegic
pMad: phosphorylated Mad
Nrx: neurexin
OE: overexpression
VNC: ventral nerve cord
BRP: bruchpilot
DLG: Discs large
Df: deficiency
CSP: cysteine string protein
SYT: synaptotagmin
GluR: glutamate receptor
AZ: active zone
PSD: postsynaptic density
SSR: subsynaptic reticulum
EJP: excitatory junction potential
mEJP: miniature excitatory junction potentials
UAS: upstream activation sequence
DSHB: Developmental Studies Hybridoma Bank.
