# Supplementary material for: Neuroligin 4 regulates synaptic growth via the bone morphogenetic protein (BMP) signaling pathway at the Drosophila neuromuscular junction
Source: J Biol Chem. 2017 Sep 14;292(44):17991–8005. doi: 10.1074/jbc.M117.810242 (PMC5672027; doi:10.1074/jbc.M117.810242)
Supplement: Supplemental Data [file supp_292_44_17991__index.html]

Neuroligin 4 Regulates Synaptic Growth via the Bone Morphogenetic Protein (BMP) Signaling Pathway at the Drosophila Neuromuscular Junction — Neuroligin 4 regulates synaptic growth via the bone morphogenetic protein (BMP) signaling pathway at the Drosophila neuromuscular junction — Drosophila neuroligin 4 regulates NMJ growth via BMP pathway — Supplemental Data 

# Neuroligin 4 regulates synaptic growth via the bone morphogenetic protein (BMP) signaling pathway at the *Drosophila* neuromuscular junction

## Supplemental Data

- Supplemental Data (.pdf, 424 KB) - Supplemental data
