# Supplementary material for: Neuroligin 4 regulates synaptic growth via the bone morphogenetic protein (BMP) signaling pathway at the Drosophila neuromuscular junction
Source: J Biol Chem. 2017 Sep 14;292(44):17991–8005. doi: 10.1074/jbc.M117.810242 (PMC5672027; doi:10.1074/jbc.M117.810242)
Supplement: Supplemental Data [file 10.1074_M117.810242_jbc.M117.810242-1.pdf]

Neuroligin 4 Regulates Synaptic Growth via the BMP Signaling Pathway at the *Drosophila* Neuromuscular Junction

Xinwang Zhang[1,3], Menglong Rui[1,2], Guangmin Gan[2], Cong Huang[1], Jukang Yi[2], Huihui Lv[1,2], Wei Xie[1,2*]

SUPPLEMENTAL DATA

Figure S1

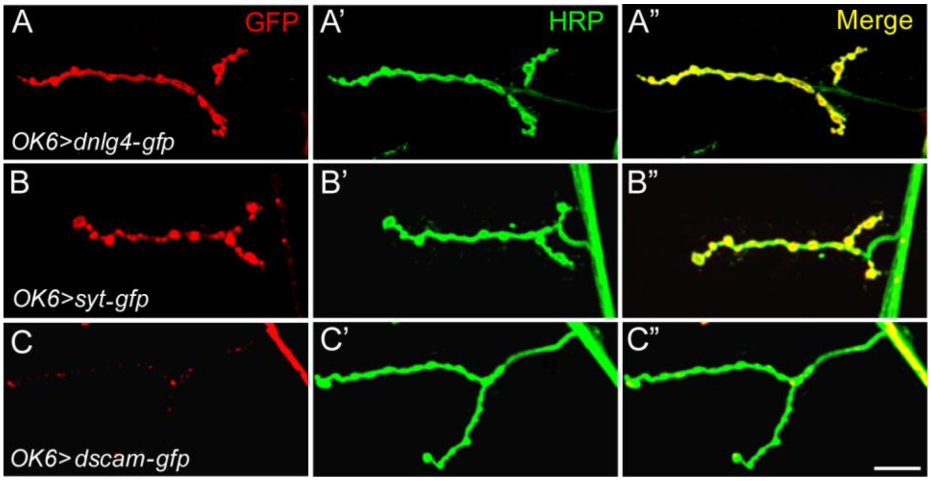

Figure S1. Motoneruon-expressed DNlg4-GFP is localized in synaptic boutons of NMJs. (A-C") The type I boutons of NMJ4 from indicated genotypes co-stained with anti-GFP (red) and anti-HRP (green) antibody, showing that DNlg4 (tagged by GFP) was localized in synaptic boutons of NMJs when it was expressed in motoneurons by a motoneuron-specific OK6-Gal4 driver (A-A"), which was same as the presynaptic molecule Syt (tagged by GFP) (B-B"). However, the postsynaptic molecule Dscam (tagged by GFP) did not locate to the synaptic boutons of NMJs when it was expressed by the same Gal4 driver. Scale bar: 20 μm.

Figure S2

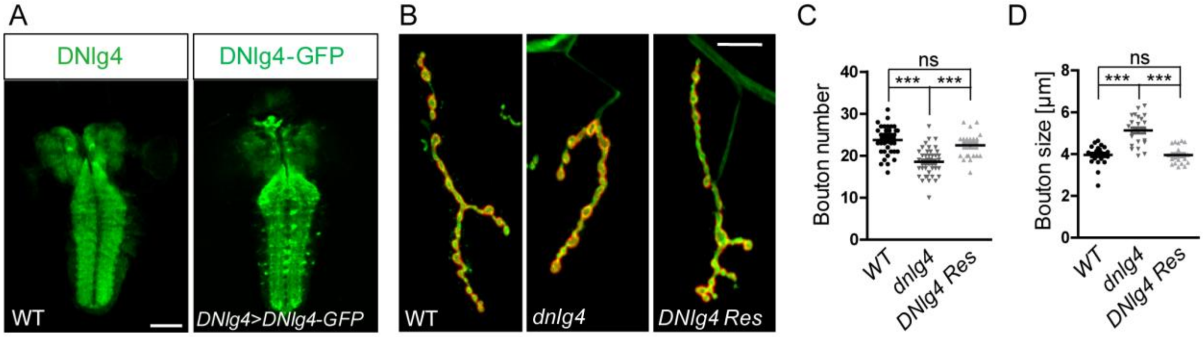

Figure S2. **Exogenous DNlg4 expressed by the DNlg4-Gal4 driver could mimic the endogenous DNlg4 in expression pattern and function.** (A) The larval brain from WT flies labeled by anti-DNlg4, and from DNlg4-GFP-overexpressing flies driven by the DNlg4-Gal4 driver (GFP is the spontaneous green fluorescence), showing that the expression pattern of DNlg4-GFP in larval brain driven by the DNlg4-Gal4 driver was similar with the expression pattern of endogenous DNlg4. (B) Representative NMJ4 morphology in WT, *dnlg4* mutant and DNlg4-Gal4 rescue flies (*dnlg4;DNlg4-Gal4>UAS-dnlg4*) (DNlg4 Res). (C, D) Quantification of the bouton number and bouton size in genotypes indicated in B, showing that the morphological defects of NMJ in *dnlg4* mutants were rescued by exogenous DNlg4 when it was expressed by DNlg4-Gal4 driver. The Mann-Whitney test was used. ***, $p < 0.001$. Scale bar, 100 μm (A); 20 μm (B).

Figure S3

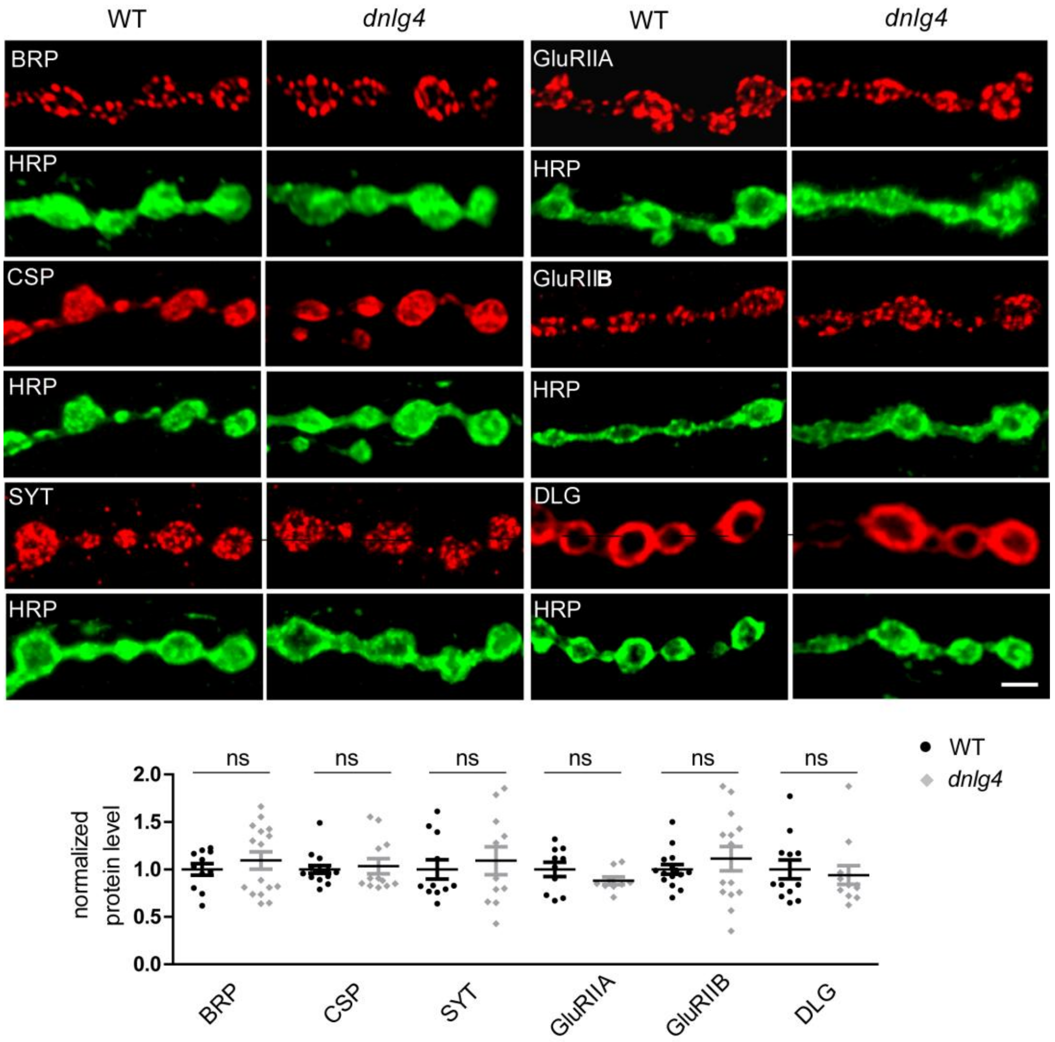

Figure S3. Several synaptic molecules are normal in *dnlg4* mutants. Synaptic boutons of NMJs from the WT and *dnlg4* mutant lines immunostained separately with anti-BRP, anti-CSP, anti-SYT, anti-GluIIA, anti-GluIIB and ant-DLG antibodies, showing that the protein levels and distribution of these molecules were not changed in the *dnlg4* mutants. HRP was labeled and used as an internal control in each case. The Mann-Whitney test was used. ns, no significance. Scale bar, 5 μm.

Figure S4

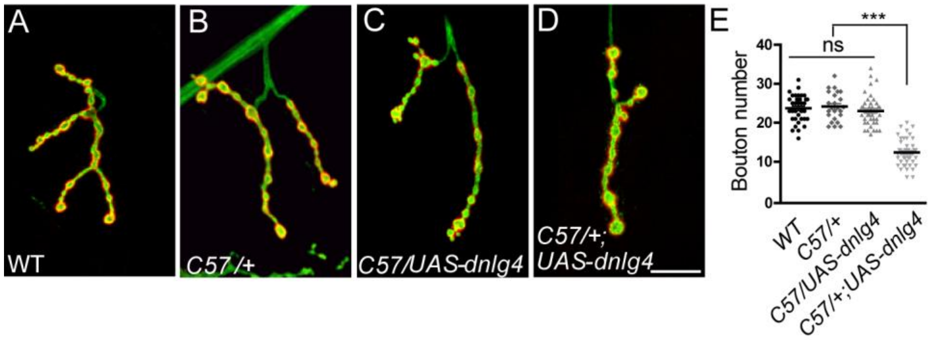

Figure S4. Postsynaptic overexpression of DNlg4 did not promote the proliferation of synaptic boutons at NMJs. (A-D) Representative NMJ4 morphology in WT, C57/+, one copy of *dnlg4*-overexpressing lines (*C57/+;UAS-dnlg4/+*) and two copies of *dnlg4*-overexpressing lines (*C57/+;UAS-dnlg4*), driven by the muscle-specific C57-Gal4 driver. (E) Quantification of the bouton number in genotypes indicated in A-D, showing that overexpression of DNlg4 postsynaptically did not induce proliferation of synaptic boutons at NMJs. Conversely, overexpression of two copies of the *dnlg4* transgene in muscle cells led to a significant reduction in bouton number. The Mann-Whitney test was used. ***, $p < 0.001$, ns, no significance. Scale Bar, 20 μm.

Figure S5

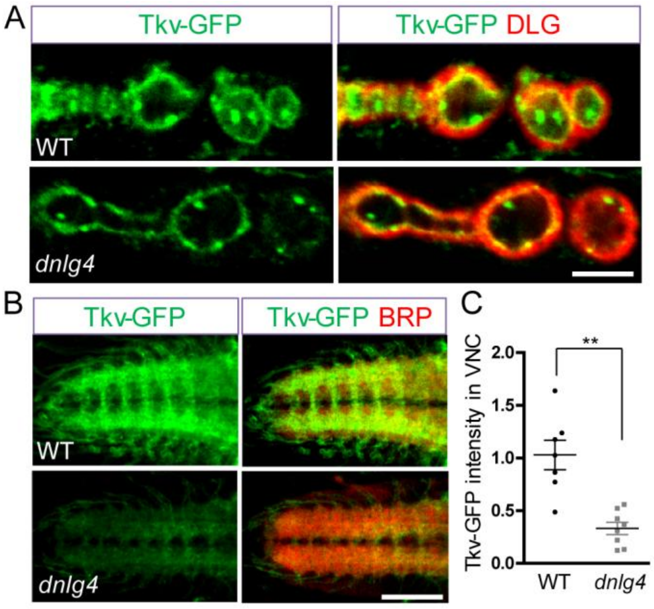

Figure S5. Tkv is decreased in *dnlg4* mutants. (A, B) Synaptic boutons of the NMJs (A) co-stained with anti-GFP (green) and anti-DLG antibody (red), and VNC sections of third-instar larval brain (B) labeled for BRP (red) (GFP is the spontaneous green fluorescence) from the controls

(*WT;Elav >UAS-Tkv-GFP*) and *dnlg4* mutants (*dnlg4;Elav >UAS-Tkv-GFP*), showing that the Tkv-GFP was decreased in the *dnlg4* mutants, and that no excess Tkv-GFP proteins accumulated in the soma of motor neurons in *dnlg4* mutants. (C) Quantification of Tkv-GFP fluorescent intensities in genotypes in B. The Mann-Whitney test was used. **, $p < 0.01$. Scale bar, 5 μm (A), 100 μm (B).

Figure S6

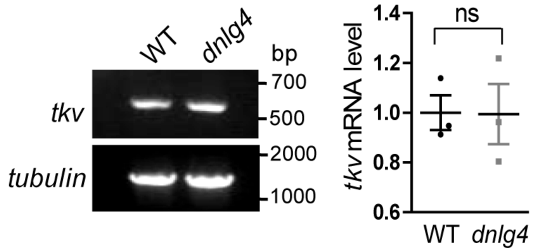

Figure S6. The mRNA level of *tkv* is not altered in *dnlg4* mutants. RT-PCR analyses of *tkv* mRNA in larval brain from the WT and *dnlg4* mutant lines showed a comparable *tkv* mRNA level in the both lines. The Mann-Whitney test was used, ns, no significance.

Text S1. Generation of the *UAS-dnlg4-EGFP* fly To generate *UAS-dnlg4-EGFP* transgenic flies, the strategy described in previous reports (1,2) was referred to. Full length DNlg4-GFP was generated by inserting GFP between amino acids L859 and Q860. The insert site is about 12 amino acids downstream of the transmembrane domain and these 12 amino acids are repeated downstream of the GFP to assure a complete intracellular domain. A PCR product of *dnlg4*-GFP with flanking EcoRI and XbaI restriction sites was cloned into the pUAST vector, and the recombinant construct was embryo injected into w^{1118} flies.

REFERENCES

1. Sheridan, D. L., Berlot, C. H., Robert, A., Inglis, F. M., Jakobsdottir, K. B., Howe, J. R., and Hughes, T. E. (2002) A new way to rapidly create functional, fluorescent fusion proteins: random insertion of GFP with an in vitro transposition reaction. *BMC neuroscience* **3**, 7
2. Dresbach, T., Neeb, A., Meyer, G., Gundelfinger, E. D., and Brose, N. (2004) Synaptic targeting of neuroligin is independent of neurexin and SAP90/PSD95 binding. *Molecular and cellular neurosciences* **27**, 227-235
